# Community referral system influencing caregiver health-seeking for childhood pneumonia in Endebess sub-county, Kenya

**DOI:** 10.4102/jphia.v16i1.801

**Published:** 2025-05-14

**Authors:** Everlyne N. Opuba, Patrick O. Onyango, Jane A. Owenga

**Affiliations:** 1 Division of Vaccines and Immunisation, Ministry of Health, Trans-Nzoia County, Nairobi, Kenya; 2 School of Physical and Biological Sciences, Maseno University, Kisumu, Kenya; 3 Department of Public and Community Health and Development, Jaramogi Oginga Odinga University of Science and Technology, Kisumu, Kenya

**Keywords:** community referral, health-seeking, caregiver, pneumonia, Kenya

## Abstract

**Background:**

Pneumonia is the primary infectious cause of mortality in children under five, with approximately 800 000 deaths annually in low-income settings. In Kenya, pneumonia accounted for 16% of child deaths in 2022. Good treatment outcome relies on efficient referral system and timely hospital access. However, monitoring referral completion remained challenging in Endebess hospitals.

**Aim:**

To assess determinants and key barriers to utilisation of community referral system.

**Setting:**

Seven public hospitals in Endebess sub-County in Kenya.

**Methods:**

This mixed-methods study involved 273 caregivers, 24 health personnel, 40 Community Health Volunteers (CHV’s) and 4 Community Health Assistants. Data were collected using questionnaires and interviews. Quantitative analysis used Statistical Package for Social Sciences Version 22 (Chi-square, logistic regression; *p* < 0.05). Qualitative data were analysed using thematic analyses.

**Results:**

Overall, 112 caregivers (41%) were referred. However, only 19 referral forms (17%) were filed at hospitals and 10 children (52.6%) recorded in service delivery logbook. Referral completion was significantly associated with distance to the hospital (*p* = 0.021), whether a CHV had accompanied the patient (*p* = 0.002) and household income (*p*= 0.040). Caregivers with self-help group savings were more likely to visit the hospital within 24 h of referral (*p* = 0.002, OR [odds ratio] = 3.8, 95% CI [confidence interval] = 1.639–8.813) than those without savings.

**Conclusion:**

Utilising CHV diaries and household registers improves referral completion, highlighting the need for digital integration to strengthen data concordance.

**Contribution:**

This study informed policymakers on strengthening community referrals by emphasising CHV report verification, mentorship on documentation and ensuring referral completion.

## Introduction

Pneumonia is the primary cause of paediatric deaths, claiming 2200 lives daily worldwide.^1^ Kenya is currently ranked among the top 15 countries with an annual mortality rate of 50.3 per 10 000 children under five.^2^ Globally, less than two-thirds of children with Acute Respiratory Infections (ARI) symptoms sought care from a healthcare provider in 2021.^3,4^ In sub-Saharan Africa, few paediatric pneumonia cases receive the care they need.^5^ Most pneumonia deaths are preventable through immunisation and low-cost treatment. However, slow progress on reducing paediatric pneumonia deaths hinders the 2030 Sustainable Development Goal (SDG) of eliminating preventable child deaths.^3,6^

The integrated Global Action Plan for the Prevention and Control of Pneumonia and Diarrhoea (GAPPD) aimed at ending preventable pneumonia deaths by 2025 and reducing pneumonia-related mortality in children under five to below 3 per 1000 live births.^7^ In addition, GAPPD aims for 90% of children with pneumonia symptoms to receive care at a health facility by 2025.^8^ Interestingly, if the current rate of slow progress continues, then Kenya is expected to reach the 2025 GAPPD target by 2029.^8^ Primary health care is the most effective and efficient approach to achieving universal health coverage.^9^ Therefore, early and appropriate care-seeking ensures timely diagnosis, treatment and referral of sick children. However, population-based survey data indicate that there has been slow progress in care-seeking for children with ARI.^10^ Progress in reducing deaths from pneumonia is essential to meeting the SDG child survival target of less than 25 child deaths per 1000 live births.^3^ Globally, only 68% of children with suspected pneumonia receive care at a health facility.^10^ Unfortunately, care-seeking for severe respiratory illness in Kenya is still low at 67%, with most caregivers seeking care when it is too late to save the sick child.^11^ Improving care-seeking requires continuous engagement with the community. Therefore, a well-functioning community-to-facility referral system is essential for saving lives and ensuring continuous high-quality care. The community and primary healthcare (PHC) services fulfil up to 90% of children’s health needs at a lower cost.^12^ The State Department of Health in Kenya aims to improve PHC uptake through involvement of Community Health Volunteers (CHVs) and community health referrals. However, community health service coverage in Kenya stands at only 59%, with some counties, including Endebess Primary Care Network (PCN) still reporting as low as 17% in 2022.^13^ The 2023–2024 sub-County data review and quality audit meetings in Endebess have continued to reveal discrepancies in reporting community referrals and facility arrivals to include gaps in utilisation of CHVs commodity packs.^14^ Existing literature from low-income countries shows that fully utilising CHVs significantly improves the uptake of PHC services.^15^ A descriptive cross-sectional study in Bangladesh identified various channels to reach households, including stakeholders’ meetings, household education, community self-help groups, CHVs accompanying patients to the facility and accessible health facility services.^16^

**FIGURE 1:**
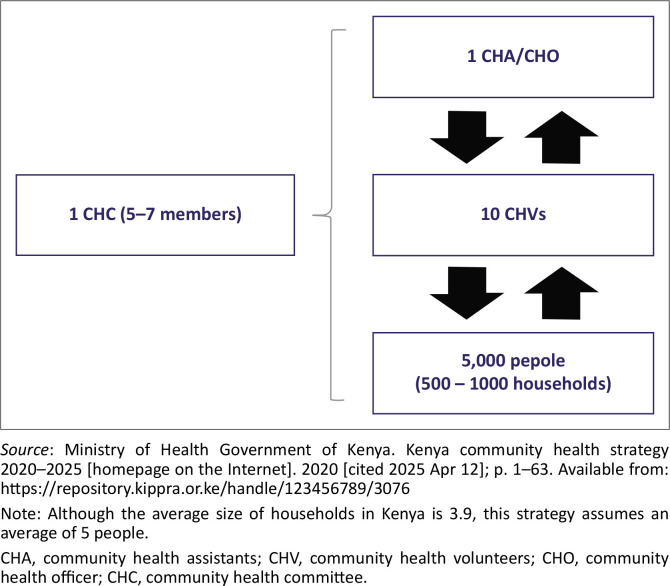
Community health unit structure in Kenya.

Community health workers (CHWs), known as CHVs in Kenya, play a key role in increasing access and utilisation of PHC services, especially in low-income settings.^17^ In Kenya, CHVs are trained to focus on health promotion through health education, disease prevention and identification of cases for referral from Community Units (CU) to health facilities.^18^ In the Kenyan setting, a CU is a collection of households served by 10 CHVs supervised by a Community Health Assistant (CHA) as illustrated in Figure 1. In this study, community referral referred to a CHV issuing a referral slip for a sick child to a health facility, with complete referrals requiring hospital feedback to the CU. This study aimed to assess key determinants, barriers and potential improvements to improve the community referral system.

## Research methods and design

### Study design and setting

A mixed-method study was conducted in Endebess Sub-County, Trans-Nzoia County, Kenya, from May to August 2019. Despite the time of data collection, recent literature still highlights gaps in community referral system, making the study findings relevant for informing policy and future practice. The study included caregivers of children with pneumonia, health personnel, CHAs and CHVs from seven public hospitals. The study area exhibited a population density of 203 persons per square kilometre, with 130 747 individuals and 21 525 households, as reported in the June 2017 Mosquito Net distribution report in Trans Nzoia County.^19,20^ It borders West Pokot, Kwanza and Saboti Sub-Counties, as well as Uganda. The primary economic activities are agriculture and tourism. The sub-County is further divided into 14 CUs with seven public health facilities. The study focused in the paediatric unit, outpatient department, specifically within the clinical officer’s consultation rooms and the registration desk.

### Study population

The study population consisted of caregivers of children under five with pneumonia seeking treatment at Endebess PCN Hospitals, either independently or through CHV referrals. Additionally, health personnel involved in pneumonia diagnosis and treatment, including 20 nurses, 3 clinicians and 1 medical officer, were included. Community Health Volunteers involved in referrals and CHAs supervising CHVs in PCNs were also part of the study population.

### Sample size determination

In the previous year, 3283 children under 5 years with clinical presentation of pneumonia were treated at health facilities across the study area. The sample size was estimated using the Cochran formula followed by a correction factor for finite populations. During the study period, the estimated proportion of children with pneumonia was 47.7%, with a 5% precision at a 95% confidence interval. The final sample size was 273 caregivers of children with pneumonia, sampled from all seven public health facilities.

### Sampling procedure

Sampling was based on the percentage contribution of each hospital to the total pneumonia cases, as shown in Table 1. To identify study participants, a straightforward simple random sampling method was employed by approaching sampled caregivers of children under 5 years diagnosed with pneumonia at hospitals in both the paediatric unit and outpatient department. Eligible children under five with pneumonia, whose caregivers gave consent were selected from the under-five register, utilising the diagnosis column within the register. Caregivers whose children had pneumonia plus other medical conditions were excluded from the study. During the study period, numbered papers were generated daily; even-numbered participants meeting the eligibility criteria were enrolled on the first day and odd-numbered participants were enrolled on the next day, continuing until the desired sample size was attained. For the qualitative component, the study encompassed all 24 healthcare professionals in the paediatric unit, all four CHAs in the sub-County and all 43 CHVs who had received training on the community component package. However, 40 (93%) CHVs were reached and interviewed because the remaining three were inactive and rarely attended meetings.

**TABLE 1:** Number of sampled caregivers within the sub-County hospitals.

S. no	Ward	Facility	Percentage contributing to pneumonia cases	Sampled caregivers
1	Endebess	Endebess Sub-County Hospital	18.73	51
		Mt. Elgon Health Centre	17.15	47
		Kitum Dispensary	15.66	43
2	Matumbei	Kaibei Dispensary	11.73	32
		Kimwondo Health Centre	12.88	35
		Matumbei Dispensary	6.15	17
3	Chepchoina	Chepchoina Health Centre	17.70	48

**Total**			**100.00**	**273**

S. no, serial number.

### Data collection procedures

Before commencing data collection, a pilot study was conducted to assess the validity and reliability of the data collection instruments through test-retest reliability. Pearson’s correlation coefficient was employed to assess the relationship between the two tests, with a reliability coefficient of ≥ 0.7 deemed acceptable (*r* = 0.950). Furthermore, the content of the data collection instrument was validated through expert opinions, critiques and advice from colleagues and peers. This research study used a semi-structured questionnaire and key informant interview guides for data collection. The questionnaire was pretested on caregivers of children with pneumonia while key informant interview guides were pretested on CHVs, CHAs and health personnel in Saboti PCN, 30 km west of Endebess PCN. Saboti was preferred because of its similar socio-demographic characteristics to Endebess.

Pretesting refined unclear questions, and each questionnaire took 30 min – 40 min to administer. Trained research assistants conducted face-to-face interviews with caregivers and key informants, and all responses were recorded through note-taking. Interviewers were briefed on the study background, significance, logic and process of sampling and thoroughly trained on the questionnaire and key informant interview guide to avoid interviewer bias. After training, a rehearsal of the interviewing process was conducted to test the questionnaire. Supervision involved spot observations of interviews with interviewee consent at various health facilities.

### Data management and analyses

The data collection tools were verified to ensure they were complete. Quantitative data were coded, edited and cleaned to identify and rectify any errors, while qualitative data were organised into tables based on emerging themes. Thematic analysis was conducted sequentially, beginning with reading through the text and taking initial notes. Next, shorthand labels were created to describe the ideas expressed in the text. Recurring patterns were then identified from these shorthand labels to generate themes. The themes were reviewed by comparing them against the data set, and adjusted as needed by splitting, combining, discarding or creating new themes. Finally, the themes were defined and named. The data were entered into an Excel worksheet and subsequently verified to ensure that subject identification numbers matched the data recorded in the questionnaire. Finally, data were imported to Statistical Package for Social Sciences (SPSS) version 22, where descriptive statistics were calculated using counts and percentages, and inferential statistical analyses were conducted. The Chi-square test of independence compared differences in distance to the health facility, household income, level of education and being accompanied by a CHV to the hospital. The Chi-square test assumed that the variables were categorical with independent observations, and expected frequencies were above five per cell but was limited to categorical data. The logistic regression model analysed the relationship between factors influencing access to healthcare and timely health-seeking upon referral, having assumed that data is from a normally distributed population (Smirnov test, *p* = 0.222) and there is a linear predictor-outcome relationship. A *p*-value of ≤ 0.05 was considered statistically significant for Chi-square and logistic regression test statistics.

### Operational definition of terms

The study’s outcome variable was health-seeking behaviour, measured by the time taken to seek care after a referral. Seeking care within 24 h was classified as prompt health-seeking, while seeking care beyond 24 h was considered delayed care-seeking.^9^

### Ethical considerations

The study received approval from the Ethical Review Board of the University of Eastern Africa, Baraton (Reference No: UEAB/06/03/2019) and a permit was granted by the National Commission for Science, Technology, and Innovation (NACOSTI/P/19/32404/29550). Caregiver written informed consent was obtained, and participants’ confidentiality was ensured by the use of unique identifiers instead of names. Interviews were conducted in separate rooms for privacy, and completed questionnaires and interview guides were securely stored under lock and key in the cupboard to maintain confidentiality. Children benefited indirectly as findings were used to improve pneumonia care. There were no potential risks involved as there was no invasive procedure done.

## Results

### Study participant demographics

Table 2 summarises participants’ socio-demographics of the 273 caregivers of children with pneumonia, 263 (96.3%) were female and 10 (3.7%) were male. Most caregivers were business people, 96 (35.2%), followed by farmers 93 (34%), unemployed 63 (23.1%) and formally employed 21 (7.7%). Most children, 147 (53.8%) were 0–12 months old and the rest 126 (46.2%) above 12 months.

**TABLE 2:** Social demographic aspects of respondents.

Characteristic	Frequency	Percentage
Gender of the caregiver		
Male	10	3.7
Female	263	96.3
Relationship to the child		
Father	10	3.3
Mother	252	92.3
Grandmother	11	4.4
Age of the caregiver (years)		
< 1	4	1.5
18–24	64	23.4
25–34	115	42.1
35–44	77	28.2
45 and above	13	4.8
Marital status		
Single	27	9.9
Married	246	90.1
Age of the child (months)		
0–12	147	53.8
13–24	47	17.2
25–36	42	15.4
37–48	19	7.0
49–59	18	6.6
Occupation		
Employed	21	7.7
Business	96	35.2
Farmer	93	34.0
None	63	23.1
Household income		
KES < 5 000	187	68.5
KES 5 000–10 000	71	26.0
KES > 10 000	15	5.5
Education level		
Never school	5	1.8
Primary	106	38.8
Secondary	137	50.2
College	23	8.4
University	2	0.8

### Perception of the community referral system

All of health personnel, 24 (100%), 4 (100%) CHAs and 112 (41%) caregivers recognised the value of services provided by CHVs and their role as a crucial link between the CUs and health facilities (Table 4). Health personnel noted that traditional beliefs including preference for home remedies, religious myths and trust in traditional healers over formal healthcare affected referral acceptance despite CHVs’ efforts in home visits and referrals. The CHAs and CHVs also pointed out that some patients lacked urgency in seeking care and some families refused nearby hospital visits.

### Communication between Community Health Volunteers, Community Health Assistants and health personnel

There was limited interaction between the CHVs and health personnel in health facilities, with most CHVs 32 (80%) visiting their link hospitals once a month during facility monthly meetings with their supervisors and the CHAs. On the reason as to why there was minimal interaction, 27 (84%) CHVs attributed it to lack of transport to hospitals because most of them were in the low-income bracket and were not given a stipend by hospitals; some 5 (16%) complained of long distance to hospitals as their homes were more than 5 km from hospitals (Table 4). However, of the minority, 8 (20%) who interacted with the facility more than once a month lived closer to their link hospital.

### Use of community referral forms

Surprisingly, 40 (100%) of CHVs lacked official referral forms and instead, they used exercise book paper as substitutes (Table 4). It was interesting to note that of the 112 (41%) referrals, only 19 (17%) forms were stored by the CHAs in the health facility and on the question as to why the forms were not filed at hospitals, health personnel cited work exigencies 18 (75%), lack of a referral file in the facility 4 (17%), CHA’s dual role in facility and community visits 2 (8%) (Table 4).

### Barriers to community referral identified by health personnel

Limitation to the use of referral data was related to lack of feedback between the health personnel and CHVs 15 (63%), high workload at hospitals 1 (4%) and documentation gaps on referral forms 5 (21%), and hospital health personnel not participating in CHVs facility monthly meetings 3 (12%) (Table 4). Health personnel also pointed out that inconsistent follow-ups by CHVs led to referral dropouts. In addition, some caregivers who prioritised work, childcare, and household duties often opted for over-the-counter medication instead of hospital visits upon referral.

### Association between factors and health-seeking behaviour upon referral

Timely access to utilisation of health care services was significantly influenced by distance to the health facility (Chi-square test of independence, *p* = 0.021), economic accessibility to health care (*p* = 0.001) and being accompanied by a CHV to hospital upon referral (*p* = 0.002) (Table 3). In addition, a logistic regression showed a significant relationship between saving money in local groups and timely health-seeking upon referral (χ^2^ 9.981, *p* = 0.002). The model also explained 11.7% of variation in time taken by caregivers to seek for care upon referral and correctly classified 68.8% of cases. Caregivers who had set aside some money were 3.8 times more likely to visit the hospital within 24 h upon referral (*p* = 0.002; odds ratio, OR = 3.8, 95% confidence interval, CI = 1.639–8.813). Over half 60 (54%) of caregivers had saved some money with self-help groups, while a few 17 (15%) saved with village-level saving groups, as shown in Table 4. Caregivers also cited the high cost of seeking care upon referral to the cost of transport to hospitals 50 (45%), the need to purchase unavailable drugs in hospitals 40 (36%) and cost of investigations in the laboratory or x-ray services 22 (19%) as shown in Table 4.

**TABLE 3:** Association between factors and health-seeking behaviour upon referral.

Variable	Time taken to seek for care				Chi-square test	*p*-value (For major categories)
	≤ 24 hrs		> 24 hrs			
	*n*	%	*n*	%		
**Distance to facility**					5.320	0.021
Time taken to walk (hours)						
≤ 1	12	36.4	21	63.6	-	-
> 1	13	16.5	66	83.5	-	-
1–2	10	-	38	-	-	-
2–3	3	-	28	-	-	-
**Economic accessibility**					10.182	0.001
Set aside resources						
Yes	57	74	20	26	-	-
No	15	42.9	20	57.1	-	-
**Accompanied by a CHV upon referral**					9.184	0.002
Yes	11	68.8	5	31.2	-	-
No	5	20.8	19	79.2	-	-
**Household income**					4.238	0.040
USD < 91.12	33	52	31	48	-	-
USD > 91.12	34	71	14	29	-	-
**Education level**					2.496	0.114
Low level	18	33	37	67	-	-
Never	6	-	13	-	-	-
Primary	12	-	24	-	-	-
High level	27	47	30	53	-	-
Secondary	9	-	17	-	-	-
College	18	-	13	-	-	-

CHV, community health volunteers.

**TABLE 4:** Actions related to health-seeking among caregivers upon referral.

Actions related to health-seeking	Frequency	Percentage
**Perception on community referral**		
**Highly recognised**		
Health personnel	24	100
Community health assistants	4	100
Caregivers	112	41
**Utilisation of referral forms**		
Used	0	0
Not used (Lacked forms)	40	100
**Reasons for failure to file referral forms at hospitals**		
Work exigencies	18	75
Lack of referral files	4	17
1 CHA required to visit CUs	2	8
**Barriers to use of referral data itself**		
Lack of feedback between health personnel and CHV	15	63
High workload at hospitals	1	4
Documentation gaps on referral forms	5	21
Health personnel not participating in CHV monthly meetings	3	12
**Reasons for high cost of healthcare**		
Cost of transport	50	45
Purchase of drugs	40	36
Cost of investigations	22	19
**Mode of savings**		
Self-help group	60	54
Village-level saving (Merry go round groups)	17	15
**Perception of preferential treatment upon referral**		
Yes	20	50
No	20	50

CHA, community health assistants; CU, community units; CHV, community health volunteers.

### Lack of preferential treatment for referral cases

Half 20 (50%) of the CHVs reported lack of preferential treatment of referred clients at the health facility in case unaccompanied by the CHV to the hospitals. In addition, out of 112 (41%) referred caregivers, 41 (37%) complained of long queues and delays before being served at the hospitals (Table 4).

## Discussion

The community referral system remains an integral part of PHC. This study investigated determinants and barriers of complete community referral influencing health-seeking behaviour in the context of paediatric pneumonia in Endebess PCN hospitals in Kenya. Out of the 273 caregivers interviewed, 112 (41.03%) had been referred by a CHV. This implies that CHVs have closer proximity to caregivers in their households than health personnel in hospitals. These findings align with a previous study in Malawi linking the increased uptake of health services when referrals were made by CHVs.^21^ Nearly all CHVs, CHAs and health personnel agreed that communication and the use of official community referral forms determine the completion of the referral. These findings are similar to outcomes from a previous study in Mozambique, which reported that stakeholders’ meetings involving the community and hospital personnel promote PHC.^22^

Continuous communication between CHVs, CHAs and health personnel was deemed essential to a strong, complete referral system. This aids in tracking clients and providing feedback on client referral status, whether they reached the link facility or not. Most CHVs, 34 (85%) reported that they rarely accompany their clients to health facilities. Therefore, they meet their supervisors, the CHAs and health personnel during their facility’s monthly review meetings. They attributed minimal interaction with the facilities to factors such as long distance to hospitals and the need to engage in their own income-generating activities, considering that CHVs received no government stipend. The availability of referral forms was considered an enabling factor to complete referral by health personnel, CHAs and CHVs. Findings are similar to studies conducted in Kenya at Kenyatta National Hospital.^23,24^ Most referred clients presented their referral forms at service delivery points without going through the routine process of triaging. The referral forms confirmed referral from CUs by respective CHVs. Nevertheless, all 40 (100%) CHVs lacked official referral forms; instead, they plucked papers from their exercise books and used them as referral forms. It was interesting to note that of the 112 referrals (41%), only a few 19 (17%) forms were stored by the CHA at the link hospital. Health personnel cited work exigencies, lack of a referral file and the CHA’s dual role in the facility and community visits as reasons for not filing the forms. This inconsistency could suggest various shortcomings, such as caregivers opting for alternative or private hospitals, drug stores, misplaced referral documents and CHVs not maintaining household registers. There was need to employ dedicated link desk personnel to manage community referrals, documentation and reporting. The government should also provide sufficient referral form booklets to facilitate the process. In reference to the use of data, all 24 (100%) health personnel acknowledged limitations on the use of referral data. A previous study that reported barriers to complete referral attributed it to the lack of use of referral data by health personnel.^21^

Regarding barriers to community referral identified by health personnel, firstly, there was lack of feedback between the health personnel and CHVs was the first barrier identified. Secondly, there was a high workload for health personnel to the extent that most of the time they did not participate in CHV’s monthly meetings and did not review data on the referral forms. Lastly, the data on the number of referrals were not well documented at health facilities, and therefore, it was difficult to map zones with similar trends for follow-up. For example, a study on perspectives in referrals and access to quality healthcare services reported chronic understaffing and poor documentation.^25^ The study findings also revealed that long distances to health facilities and lack of economic empowerment were barriers to seeking timely health care upon referral from the community. Most caregivers who walked for more than 2 h to a health facility often delayed seeking care. Besides, the long distance to the hospital and the bad road terrain during the rainy season were also a barrier. Regarding economic empowerment, most caregivers with emergency savings and household income above KES 10 000 (USD 91.12) sought timely care, unlike those without savings. Key demand side – barriers to referral completion in Kenya include lack of money for care and, on the supply side, long distance to health facilities.^26^ The clients who had been referred expected preferential treatment to avoid long waiting times at the health facility. Unfortunately, most health personnel did not prioritise referred clients if not accompanied by a CHV; instead, they allowed them to follow the normal process of triaging and assessment. The findings have been reported in similar settings.^27^ There is need for hospital in-charges to strengthen the referral system and establish feedback mechanisms.

This study’s limitations stem from insufficient documentation of the clients referred by CHVs to health facilities, leading to limited data on referral trends within the community. Proper documentation of referral data would help identify high-referral settlements indicating health gaps to be targeted for community dialogue and action days. As the study was conducted in all public hospitals in the sub-county, its findings may not be generalisable to private health facilities. The study was also limited to 24 health personnel because of chronic staffing levels at rural hospitals, and therefore, all 24 health workers were included in the study. Nevertheless, the CHVs demonstrated effective referral practices to health facilities, and it became apparent that hospitals underutilised the referral information provided.

## Conclusion

Complete referral relied on effective communication among CHVs, CHAs and health personnel, as well as use of referral forms by CHVs. Health personnel identified a limitation in the use of referral data itself as a barrier to community referral. Three major themes emerged from the present study as barriers to referral from the community to hospitals, first being distance to the health facility. The caregivers most significantly affected were those residing within walking distance of more than 2 h from their designated link hospitals. This could be attributed to firstly, lack of financial resources to cover the expenses associated with transportation to the hospital. Secondly, financial constraints hindered caregivers’ access to healthcare. Thirdly, the absence of preferential treatment for referral cases by CHVs in link hospitals posed a setback to community referral. This study concludes that incomplete referrals and poor documentation may undermine the good efforts being made by the CHVs within the community. The study findings also highlight that equitable health access requires regular mobile clinics and CHV-led outreach, including diagnosis, care and treatment beyond preventive health care, to address transport barriers in hard-to-reach areas. Therefore, it is recommended that link hospitals synthesise and utilise referral data effectively by conducting monthly facility-community data review meetings and share findings in CHVs’ facility monthly meetings. Review of community data may assist health personnel to focus on interventions to promote health care among community members and inform policymakers on training needs to address the identified gaps. Future studies on the effectiveness of CHVs and CHA monthly meetings and facility-community data reviews on timely uptake of preventive and promotive health services should be considered.
